# Hepatitis B virus infection and replication in human bone marrow mesenchymal stem cells

**DOI:** 10.1186/1743-422X-8-486

**Published:** 2011-10-31

**Authors:** Ruiping Ma, Quantai Xing, Lihua Shao, Dakun Wang, Qingzhi Hao, Xia Li, Lintao Sai, Lixian Ma

**Affiliations:** 1 Department of Infectious Diseases, Qilu Hospital, Shandong University, Wenhua Xi Road 107, Jinan 250012, Shandong Province, China; 2 Department of Laboratory Sciences, School of Public Health, Shandong University, Wenhua Xi Road 107, Jinan 250012, Shandong Province, China; 3 Cryo Medicine Laboratory, Qilu Hospital, Shandong University, Wenhua Xi Road 107, Jinan 250012, Shandong Province, China; 4 Department of Peripheral Vascular, Affiliated Hospital of Shandong University of Traditional Chinese Medicine, Wenhua Xi Road 42, Jinan 250011, Shandong Province, China; 5 Laboratory for Tumor Immunity and Traditional Chinese Drug Immunity, Institute of Basic Medicine, Shandong Academy of Medical Science, Jingshi Road 89, Jinan 250062 Shandong Province, China

**Keywords:** Hepatitis B virus, Infection, Replication, Human bone marrow mesenchymal stem cell, Host Cell

## Abstract

**Background:**

Hepatitis B virus (HBV) infection is a blood borne infectious disease that affects the liver. Human bone marrow mesenchymal stem cells (BMSCs) may serve as a cell source for adult stem cell transplantation in liver repair. However, the susceptibility of human BMSCs to HBV infection is poorly understood. The aim of this study was to investigate the infection and replication of HBV in cultures of human BMSCs.

**Results:**

Human BMSCs were confirmed using flow cytometry. Intracellular HBV DNA was detected at d 2 after infection and maintained at relatively high levels from d 6 to d 12. The maximal level of intracellular HBV DNA was 9.37 × 10^5 ^copies/mL. The extracellular HBV DNA was observed from d 3 to d 15, and the levels ranged from 3.792 × 10^2 ^copies/mL to 4.067 × 10^5 ^copies/mL. HBsAg in the culture medium was detected from d 2 to d 16. HBeAg secretion was positive from d 5 to d 13. HBcAg constantly showed positive signals in approximately 7%-20% of BMSCs from 2 days after exposure. Intracellular HBV covalently closed circular DNA (cccDNA) could be detected as early as 2 days postinfection, and strong signals were obtained with increasing time.

**Conclusion:**

HBV can infect and replicate in human BMSCs. Human BMSCs may be a useful tool for investigating HBV life-cycle and the mechanism of initial virus-cell interactions.

## Background

Hepatitis B is one of the most common infectious diseases worldwide. It has been estimated that 2 billion people have been infected with hepatitis B virus (HBV). In addition, 360 million people have chronic HBV infection, and 0.6 million people die each year from HBV-related liver disease or hepatocellular carcinoma [1]. Despite the existence of a preventative vaccine, HBV represents a substantial threat to public health [1]. A convenient in vitro assay for HBV natural infectivity is currently unavailable, and the early steps of the viral life cycle are not well understood. Primary human hepatocytes are susceptible to HBV [2,3]. However, the use of this model is hampered by limited resources and the technical difficulties that are associated with primary hepatocyte cultures. In recent years, liver-related stem cells have attracted intense attention due to their proliferative capabilities and inherent characteristics. Previous studies have shown that human bone marrow mesenchymal stem cells (BMSCs) can differentiate into functional hepatocyte-like cells in vitro [4,5], and restore liver function in animal models of liver failure [6,7]. However, the susceptibility of human BMSCs to HBV infection is poorly understood. In the present investigation, we demonstrated for the first time that human BMSCs fully supported the fastidious HBV infection, replication, expression, and secretion. Human BMSCs offers a new opportunity for basic research of the HBV life cycle and the mechanism that mediates the early stages of virus-cell interactions.

## Methods

### Isolation and culture of human BMSCs

Under a protocol that was approved by the Ethics Committee of Shandong University, human bone marrow was aspirated from the iliac crest of healthy donors (18-36 years) with their informed consent. All donors had no serologic evidence of hepatitis or previous HBV infection. The mononuclear cell fraction was separated via centrifugation using a Ficoll-Paque gradient and plated at 1 × 10^5 ^cells/cm^2 ^in low-glucose DMEM (Gibco) that was supplemented with 10% FBS (Gibco) and 100 IU/mL penicillin and 100 mg/mL streptomycin. The cells were cultured at 37°C in a humidified atmosphere with 50 mL/L CO_2 _in air. After 3 days, the nonadherent cell fraction was removed by washing with PBS. Monolayers of attached cells were cultured until they reached 70-90% confluence. The cells were passaged five times prior to further analysis to ensure the removal of contaminating hematopoietic cells.

### Flow cytometric analysis

For cell-surface antigen phenotyping, fifth-passage BMSCs were detached and stained with phycoerythrin (PE)-conjugated monoclonal antibodies against CD45 and fluorescein isothiocyanate (FITC)-labeled antibodies against CD34, CD105 and CD90 and analyzed using flow cytometry with a FACScan (Becton Dickinson, USA). All antibodies were purchased from Becton Dickinson. Isotype control experiments were run in parallel using the same concentration of each antibody.

### Cell lines

The HepG2.2.15 cell line, which is a stable human hepatocellular carcinoma cell line that is permanently transfected with HBV-DNA, was obtained from the China Center for Type Culture Collection. HepG2.2.15 cells were cultured with high-glucose DMEM (Gibco) that was supplemented with 10% FBS, 100 IU/mL penicillin, 100 mg/mL streptomycin, and 380 ng/mL G418 in an incubator with 95% humidity and 50 mL/L CO_2 _at 37°C. HepG2.2.15 cells were used as a positive control.

### Infectious serum source

A serum sample for infection was obtained from a hepatitis B patient who was positive for HBsAg, HBeAg, and HBcAb (HBV core antibody) detection and had a HBV DNA serum load that was 5.4 × 10^8 ^copies/mL. The patient had received no antiviral therapy prior to the study and was not infected with HCV or HIV. The sera were stored at -80°C until use.

### In vitro infection

The fifth generation of BMSCs was seeded in six-well culture dishes. FBS was omitted from the media for 24 h. The cells were incubated with L-DMEM and 10% HBV sera concentration. Following 24 h of exposure, the cells were washed 5 times with PBS to remove the unabsorbed virus. PBS that was used in the sixth wash was collected for detection of HBV DNA using PCR. Thereafter, the cells were cultured with complete media and the start of this incubation was taken as time zero. We used one-well of cells without HBV serum as a negative control.

### Analysis for intracellular and extracellular HBV DNA

After infection, aliquots of the BMSCs were harvested using trypsin at various times and their culture supernatants were collected in order. All samples were stored at -80°C. The total DNA was extracted from harvested cells and culture medium according to the method described by Klintschar et al [8]. The HBV PCR fluorescent quantitative detection kit (Piji Biotec, Shenzhen, China) was used according to the manufacturer's protocols.

### Detection of HBsAg and HBeAg

The HBsAg and HBeAg levels in the media of infected BMSCs were measured using electrochemiluminescence (ECL) with the Elecsys 2010 fully-automated ECL analyzer (Roche).

### Detection of HBcAg in infected BMSCs

Infected and control cells were fixed in 40 g/L paraformaldehyde for 20 min at 4°C and permeabilized in Triton X-100 (0.5% in PBS). After blocking in goat serum (1:10 dilution in PBS) for 30 min at 37°C, the cells were incubated overnight at 4°C with a specific mouse monoclonal antibody against HBcAg (1:200 dilution) (Millipore). The secondary antibody was a FITC-tagged goat anti-mouse IgG (1:100 dilution) (SouthernBiotech), which was incubated with the cells at 37°C for 30 min. Finally, the cells were incubated for 10 minutes at room temperature in DAPI (0.5 μg/mL in PBS) and observed using an inverted fluorescence microscope (Olympus, Japan). HepG2.2.15 cells were used as a positive control. BMSCs that were not exposed to HBV were used as a negative control.

### Detection of covalently closed circular DNA (cccDNA)

Episomal DNAs, including HBV cccDNA were extracted as previously described [9]. Briefly, the cells were lysed in lysis buffer (50 mM Tris hydrochloride, 10 mM EDTA, and 1% sodium dodecyl sulfate). The protein-detergent complexes were precipitated using 2.5 M KCl (0.25volume). The lysates were shaken and centrifuged to remove the insoluble material. The supernatant, which contained viral cccDNA, was used to extract DNA using an equal volume of phenol. Nucleic acids were precipitated using 2 volumes of ethanol. Further purification of cccDNA from contaminating single- and double-stranded DNA was performed using the method of Yang [10]. The purified cccDNA samples were subjected to electrophoresis in 1% agarose, transferred to a nitrocellulose filter, and analyzed by Southern blotting using a DIG-labeled whole HBV DNA probe. The DIG High Prime DNA Labeling and Detection Starter Kit I was purchased from Roche, and the hybridization was performed according to the manufacturer's instructions.

### Statistical analysis

The data of HBV DNA, HBsAg, and HBeAg were expressed as mean ± standard deviation (SD). The results were analyzed using descriptive statistics. The statistical analysis was performed using SPSS 13.0 statistic software (SPSS Inc., USA).

## Results

### Characterization of BMSCs

**Figure 1:**
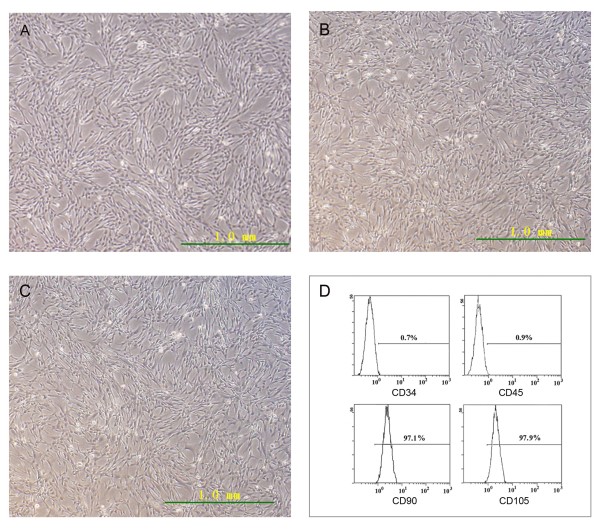
**Characterization of human BMSCs**. (A) Morphology of the third generation of human BMSCs under light microscope. (B) Morphology of the eighth generation of infected human BMSCs under light microscope. (C) Morphology of the eighth generation of uninfected human BMSCs under light microscope. (D) Analysis by flow cytometry showed that human BMSCs at passage 5 were negative for the expression of CD34 and CD45, but positive for the expression of CD90 and CD105.

Isolated human bone marrow mononuclear cells were cultured in growth media. The attached cells were morphologically similar to fibroblasts and exhibited characteristic spindle-shaped fusiform morphology (Figure 1A). Flow cytometric analysis of the cells from the fifth subculture demonstrated constitutive expression of CD105 (SH2) and CD90, but absence of CD45 and CD34, which are specific markers to hematopoietic stem cells (Figure 1D). These results are consistent with the expression pattern of surface antigens on classical BMSCs, which has been reported [6,11]. Therefore, we concluded that the sample of cells that had been cultured consisted solely of BMSCs. After infection, BMSCs were passaged every 6 days (1:3 dilution) via trypsinization. There were no obvious differences between infected BMSCs and uninfected BMSCs (Figure 1B, C). Human BMSCs cultures that were sustained for 10 weeks maintained their characteristic morphology.

### Analysis for HBV-DNA in media and cultured cells

**Figure 2:**
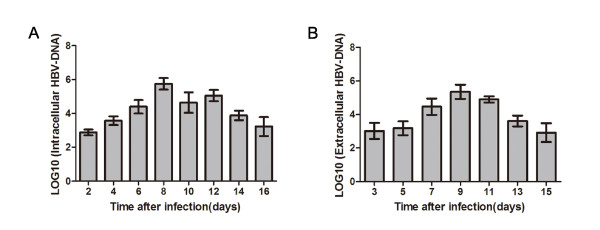
**Analysis of HBV DNA by FQ-PCR**. (A) Intracellular HBV DNA was extracted from infected human BMSCs at various times and not detected until 2 days. (B) Extracellular HBV DNA was extracted from cell supernatants at various times and not detected until 3 days. No HBV DNA was detected in the cells or supernatants at time zero (data not shown).

The intracellular HBV DNA was first detected at d 2 after infection. Relatively high levels of intracellular HBV DNA were maintained from d 6 to d 12 (Figure 2A). HBV DNA was secreted into the culture media from d 3 to d 15, with a secretion peak at d 9 (Figure 2B). Intracellular HBV DNA levels ranged from 4.750 × 10^2 ^copies/mL to 9.37 × 10^5 ^copies/mL, whereas extracellular HBV DNA levels ranged from 3.792 × 10^2 ^copies/mL to 4.067 × 10^5 ^copies/mL. These DNA molecules were not remnants of the infecting virions because no HBV DNA was detected in the cells or media at time zero.

### Detection of HBsAg and HBeAg

**Figure 3:**
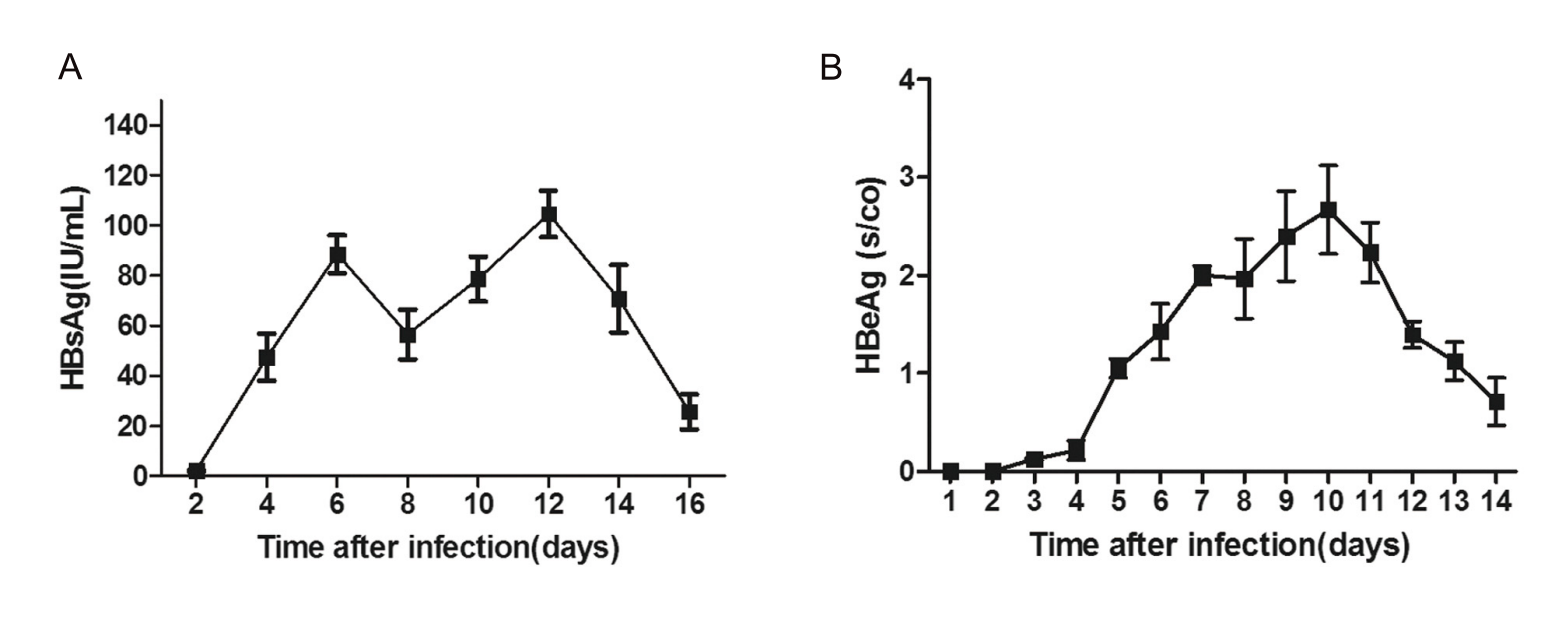
**Detection of HBsAg and HBeAg using ECL**. (A) HBsAg levels in supernatant (≥1 IU/mL was considered positive). (B) HBeAg levels in supernatant [≥1 s/co (absorbance rate/cut-off ratio) was considered positive].

The culture media were periodically collected after infection for ECL analysis of HBsAg and HBeAg levels. As shown in Figure 3A, HBsAg was detected from d 2 to d 16. The HBsAg levels ranged from 1.612 to 118.100 IU/mL (≥1 IU/mL was considered positive). HBeAg appeared positive at d 5, increased until d 10, and decreased thereafter. The HBeAg levels ranged from 0.115 to 3.407 s/co (absorbance rate/cut-off ratio) (≥1 s/co was considered positive) (Figure 3B). The samples of culture media from d 0 to d 1 were negative for HBsAg and HBeAg, indicating that these proteins were synthesized de novo and did not represent input viral antigens that were present in the inoculum.

### Expression of HBcAg

**Figure 4:**
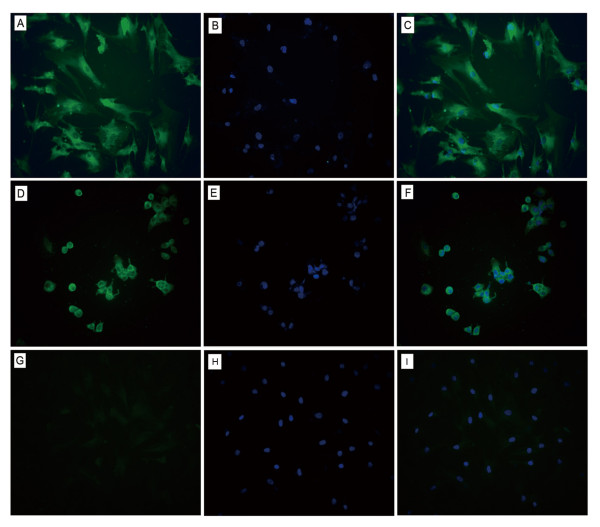
**Detection of HBcAg using indirect immunofluorescence (×200)**. (A) HBcAg in infected human BMSCs shown by FITC staining (green color). (B) The cellular nuclei in infected human BMSCs were stained with DAPI (blue color). (C) Overlaid image of images from panels A and B. (D) HBcAg in HepG2.2.15 cells (positive control) shown by FITC staining. (E) DAPI staining for nuclei of the same field as in panel D. (F) Overlaid image of images from panels D and E. (G) HBcAg in uninfected human BMSCs (negative control) shown by FITC staining. (H) DAPI staining for nuclei of the same field as in panel G. (I) Overlaid image of images from panels G and H.

To estimate the percentage of infected human BMSCs, indirect immunofluorescence was used to assay HBcAg. HBcAg expression in infected BMSCs was first detected 2 days after infection when approximately 7% of the cells were stained. Thereafter, the number of cells expressing HBcAg increased. Approximately 20% of cells expressed HBcAg at 9 days after infection. As shown in Figure 4, a diffuse but strong green fluorescence was predominantly observed in the cytoplasm of BMSCs that were infected with HBV but was occasionally observed in their nucleus. By contrast, no HBcAg was detected in uninfected BMSCs.

### HBV cccDNA detection

**Figure 5:**
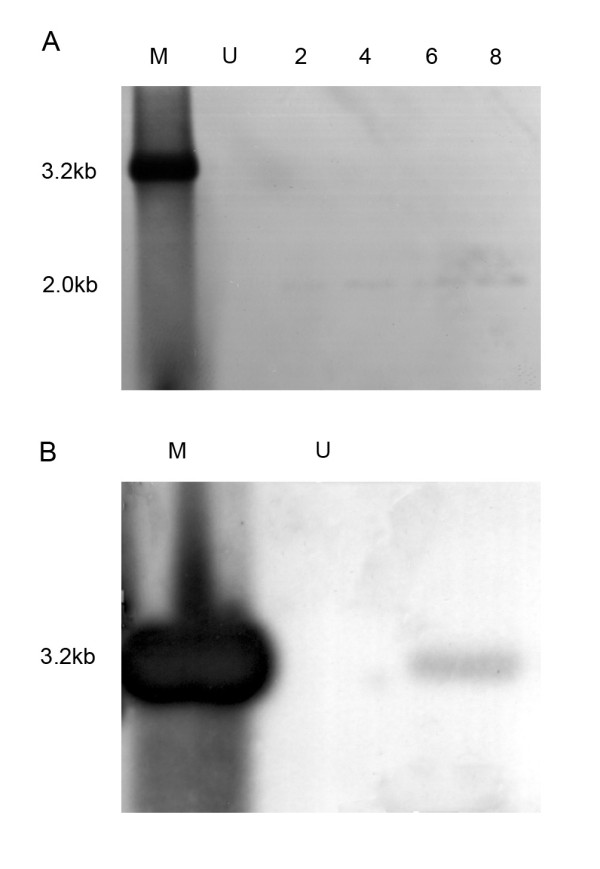
**Southern blot analysis of HBV cccDNA in infected human BMSCs**. (A) HBV cccDNA was extracted from human BMSCs at 2, 4, 6, and 8 days after infection and subjected to Southern blot hybridization. DIG-labeled whole HBV DNA probe served as a positive control (lane M). Uninfected BMSCs served as a negative control (lane U). DNA sizes are indicated on the left (kb). (B) The cccDNA cleaved by *E*coRI was analyzed using Southern blot analysis.

HBV cccDNA was prepared from the cultured BMSCs at 2, 4, 6, and 8 days after infection and analyzed using Southern blotting, when the levels of intracellular HBV-DNA increased over time. As shown in Figure 5A, a slight band of 2.0 kilobases (kb) was observed at d 2 and corresponded to the expected position for the HBV cccDNA. Stronger signals were obtained with increasing time postinfection. To further verify the presence of cccDNA, the extracted cccDNA sample was digested using the restriction enzyme *Eco*RI before loading. After digestion, the putative cccDNA band shifted to a 3.2-kb position (Figure 5B), which represents the linear double-stranded HBV genome. These results unequivocally show that HBV cccDNA is detected in infected BMSCs. By comparison, no cccDNA was detected in uninfected BMSCs.

## Discussion

Currently, the hepatoma-derived cell line is a well-established and successful system that has been used for the in vitro study of HBV. However, the viral genome is introduced by integration or transfection rather than infection [12,13]. Therefore, these cell lines are unsuitable for studying the mechanism of the early stages of virus-cell interactions, including viral attachment, penetration, and uncoating. Primary human hepatocytes allow natural penetration and full viral replication. However, the use of primary human hepatocytes is hampered by limited resources and technical difficulties that are associated with human hepatocyte culture. Treatment of primary hepatocytes with DMSO or PEG can enhance and prolong HBV infection [2,3], but these chemicals may induce artificial mechanisms for viral entry. Therefore, an ideal cell system for the study of HBV in vitro is needed. In the present study, an HBV-infected human BMSCs culture system was established using the method of co-cultivation of BMSCs with HBV-positive serum in vitro.

Several methods have been used to isolate BMSCs, including plastic adherence, gradient density centrifugation and immunomagnetic selection. The number of MSCs in the bone marrow is small. There are approximately 2 to 5 cells that are present in every 1 × 10^6 ^mononuclear cells [14]. The cells that are selected from the human bone marrow mononuclear cell fraction by plastic adherence display typical features of BMSCs [6]. Therefore, we combined the gradient density centrifugation with plastic adherence to isolate human BMSCs and to obtain abundant and pure BMSCs. In addition, flow cytometric analysis further confirmed human BMSCs identity.

In the present study, initial evaluation of the infection system was performed using quantitative fluorescence PCR (FQ-PCR) for HBV DNA. Previous studies of bone marrow cells have provided insufficient information about the quantitative analysis of HBV-DNA in culture supernatants and infected cells. FQ-PCR is highly sensitive and permits the use of small amounts of material. The FQ-PCR method that was used in this study could detect as few as 10^2 ^HBV genomes per assay. In our experiment, the levels of the intracellular and extracellular HBV-DNA were assayed during the culture period. No HBV-DNA was detected in the cells or media at time zero. Therefore, these DNA molecules could not be carryovers of the infecting virions. Viral DNA in the medium was not detected until 3 days after infection. Therefore, the appearance of HBV DNA in the medium of infected BMSCs was the result of de novo viral gene product synthesis. In contrast, no HBV-DNA was detected in uninfected BMSCs. These date indicate that HBV could infect the cultured human BMSCs and replicate in them in vitro.

In addition to assay of HBV DNA, secretory HBV antigens were identified using ECL. HBsAg represents a multi-functional activity that plays a critical role in the binding of HBV and target cells [15]. The expression of HBeAg is closely linked to HBV DNA replication [16]. Therefore, our detection of the two HBV-specific antigens provided evidence that replication and expression of the HBV genome occurred in infected BMSCs. Moreover, the efficiency of HBV infection of BMSCs in vitro, based on the expression of the HBcAg, was comparable with the infection of primary human hepatocytes [16] and human hepatoma cell lines [12,17]. HBcAg forms the capsid of viral particles and is essential for viral genome DNA replication. Our results suggest that HBV can replicate actively in cultured human BMSCs.

In order to provide further supporting evidence that HBV can infect and replicate in human BMSCs, we analyzed the intracellular HBV cccDNA via Southern blot hybridization. In our experiment, intracellular cccDNA was detected in infected BMSCs as early as 2 days postinfection. This phenomenon is consistent with the view that the appearance of cccDNA in the nucleus is one of the earliest events in the replication of hepadnaviruses [18]. The cccDNA is thought to be the template for transcription of mRNAs and pregenome RNA, which for synthesis of new DNA genomes via reverse transcription [9]. Therefore, our experimental infection system not only surports HBV infection but also reproduces the complete cycle of viral replication. Nevertheless, Southern blot analysis of total DNA isolated from BMSCs failed to demonstrate the presence of the relaxed circular and single stranded forms of HBV DNA (data not shown). This was not surprising due to the relatively low titer of HBV DNA (10^2^-10^5 ^copies/mL) as measured in the infected BMSCs using FQ-PCR. Similar results in primary human hepatocytes in vitro have been reported previously [2]. This may be due to a different host environment in culture compared to hepatocytes in vivo.

In our study, we also performed reinfection experiments. The progeny virions were infectious (data not shown). The media of infected BMSCs were incubated with mouse antibodies directed against HBsAg (anti-HBsAg). The media were assayed for infectivity using FQ-PCR and ECL. The anti-HBsAg antibodies reduced the infectivity (data not shown). These results suggest that uptake of HBV into BMSCs follows the authentic entry pathway and infected BMSCs can synthesize and secrete infectious virions into the media.

In our experiment, the infected BMSCs were sustained for 10 weeks or more and maintained their characteristic phenotype. As a subset of bone marrow stem cells, BMSCs are relatively easily separated and grown in culture. Due to their excellent proliferation capabilities without losing their initial characteristics, they can overcome the limitations associated with human hepatocyte culture (e.g., scarce resources and technical difficulties). Previous studies have indicated that human BMSCs are capable of differentiating into functional hepatocyte-like cells in vitro [4,5]. In our studies, we found that HBV could also infect and replicate in the hepatocyte-like cells induced from human BMSCs (data not shown). Interestingly, hepatocyte-like cells from human BMSCs were hard to maintain in cultures and had poor proliferative potential in vitro. These features are similar to adult human hepatocytes. The results of our study demonstrate that human BMSCs display a better advantage over BMSC-derived hepatocyte-like cells in studies of HBV.

It was reported that HBV could infect and replicate in bone marrow cells in vivo [19]. As a subset of bone marrow cells, it remains to be seen whether BMSCs are permissive for productive HBV replication in vivo. In addition, it remains unclear if HBV uses the same pathway/mechanism to infect BMSCs and hepatocytes. These need to investigate in future studies.

## Conclusion

In conclusion, several lines of evidence indicate that the human BMSCs can maintain HBV infection in vitro and support the replication of HBV DNA. Our experimental in vitro infection system will be a powerful tool to analyze the complex interactions between HBV and its host cell at the molecular levels and to evaluate different antiviral strategies. Furthermore, our findings with this system provide evidence that HBV exhibits a wider host range than previously reported, and may help explain HBV reinfection in recipients of liver transplants.

## List of abbreviations

**HBV**: Hepatitis B virus; **BMSCs**: Bone marrow mesenchymal stem cells; **cccDNA**: covalently closed circular DNA; **PE**: Phycoerythrin; **FITC**: Fluorescein isothiocyanate; **ECL**: Electrochemiluminescence; **FQ-PCR**: Quantitative fluorescence PCR.

## Competing interests

The authors declare that they have no competing interests.

## Authors' contributions

RM performed the experiments, participated in the design of the study and wrote the initial draft of the manuscript. LS, XL and DW helped to carry out the experiments. QX and QH participated in the design of the study and helped to analyze the data. LS and LM supervised, helped to design the study and finally edited the manuscript. All authors read and approved the final manuscript.
