# Correction: Hepatitis B virus infection and replication in human bone marrow mesenchymal stem cells

**DOI:** 10.1186/1743-422X-9-87

**Published:** 2012-05-04

**Authors:** Ruiping Ma, Quantai Xing, Lihua Shao, Dakun Wang, Qingzhi Hao, Xia Li, Lintao Sai, Lixian Ma

**Affiliations:** 1 Department of Infectious Diseases, Qilu Hospital, Shandong University, Wenhua Xi Road 107, Jinan 250012, Shandong Province, China; 2 Department of Laboratory Sciences, School of Public Health, Shandong University, Wenhua Xi Road 107, Jinan 250012, Shandong Province, China; 3 Cryo Medicine Laboratory, Qilu Hospital, Shandong University, Wenhua Xi Road 107, Jinan 250012, Shandong Province, China; 4 Department of Peripheral Vascular, Affiliated Hospital of Shandong University of Traditional Chinese Medicine, Wenhua Xi Road 42, Jinan 250011, Shandong Province, China; 5 Laboratory for Tumor Immunity and Traditional Chinese Drug Immunity, Institute of Basic Medicine, Shandong Academy of Medical Science, Jingshi Road 89, Jinan 250062, Shandong Province, China

## Correction

After publication of this work [1], we noted that D of Figure 1 was incorrect. Now the correct figure has been provided with this correction.

**Figure 1:**
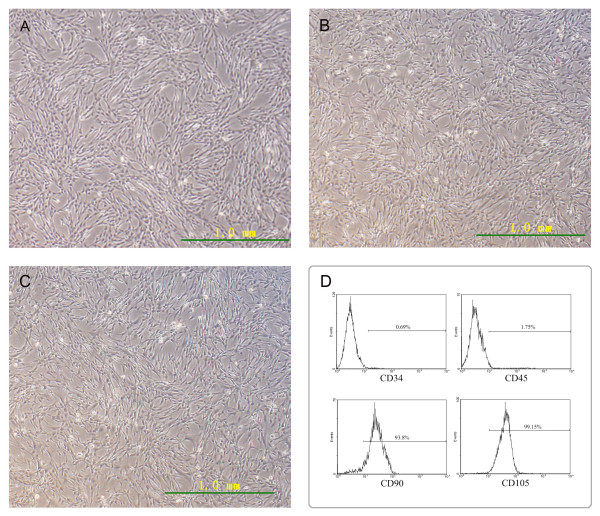
**Characterization of human BMSCs.** (**A**) Morphology of the third generation of human BMSCs under light microscope. (**B**) Morphology of the eighth generation of infected human BMSCs under light microscope. (**C**) Morphology of the eighth generation of uninfected human BMSCs under light microscope. (**D**) Analysis by flow cytometry showed that human BMSCs at passage 5 were negative for the expression of CD34 and CD45, but positive for the expression of CD90 and CD105.

## Author details

^1^Department of Infectious Diseases, Qilu Hospital, Shandong University, Wenhua Xi Road 107, Jinan 250012, Shandong Province, China. 2Department of Laboratory Sciences, School of Public Health, Shandong University, Wenhua Xi Road 107, Jinan 250012, Shandong Province, China. 3Cryo Medicine Laboratory, Qilu Hospital, Shandong University, Wenhua Xi Road 107, Jinan 250012, Shandong Province, China. 4Department of Peripheral Vascular, Affiliated Hospital of Shandong University of Traditional Chinese Medicine, Wenhua Xi Road 42, Jinan 250011, Shandong Province, China. 5Laboratory for Tumor Immunity and Traditional Chinese Drug Immunity, Institute of Basic Medicine, Shandong Academy of Medical Science, Jingshi Road 89, Jinan 250062, Shandong Province, China.
